# "Not Everything That Is Faced Can Be Changed, but Nothing Can Be Changed Until It Is Faced": A Response to Recent Commentaries

**DOI:** 10.15171/ijhpm.2017.30

**Published:** 2017-03-01

**Authors:** Solomon R. Benatar

**Affiliations:** ^1^University of Cape Town, Cape Town, South Africa.; ^2^Dalla Lana School of Public Health, University of Toronto, Toronto, ON, Canada.

I am appreciative of the responses from thoughtful colleagues to my recent article,^1^ and their support for the central role of the global political economy and my criticisms of the current approach to poverty.

Gorik Ooms and colleagues make the point that, contrary to my claim, many global scholars are indeed thinking creatively and imaginatively outside the box.^2^ Ron Labonté affirms this and includes most members of the Lancet-Oslo Commission, but acknowledges that lateral thinking is obstructed and undermined in an era of powerful social hierarchies of power and privilege, characterized by autocratic and right populist thinking.^3^ Under these circumstances the weakness of the Commission’s recommendations can in part be explained by the statement from Ooms et al that criticisms of entrenched dogma are suppressed by the covert (obstructive) workings of the academic community. The effects of long-standing neglect of historical social forces adversely affecting academic and scientific freedom,^4,5^ and absence of discussion on the genesis of structural power,^6^ are aggravated by expanding links between universities and the medical-academic-corporate-foundation industries.^7-9^ Added to this is the chilling impact of self-censorship within a ‘tyranny of silence’ associated with political correctness,^10^ and coercion through government bullying, as in Canada recently.^11^

Labonté acknowledges that we agree on today’s global crises being located within the pathology of our current global political economy and its supporting hegemonic discourse as discussed by others previously,^12-17^ and explicated in more detail by Stephen Gill.^18^ Labonté also avers that neither of us in our recent articles get sufficiently close to how change could be achieved. This point has merit, although it should be acknowledged that some suggestions have indeed been made for moving forward.^19,20^ Sir Anthony Atkinson’s proposals for addressing inequality through progressive taxation,^21^ are supported by Thomas Piketty’s recommendations for rectifying the perversities associated with the accumulation and distribution of capital.^22^ Gabriel Zucman’s detailed revelations of the massive extent of tax avoidance and evasion, with vast wealth sequestered in tax havens,^23^ together with the Panama Papers^24^ provide additional insights into how structurally determined inequalities could be rectified, at least in part. Achieving wider extension of consensus on such issues is the first important step towards exploring complex new ways of moving forward.

Ooms et al also correctly allude to the ‘elephant in the room’ as the failure to admit that some redistribution between countries is an inevitable step towards ameliorating environmental unsustainability. They concede it should be widely admitted that consumption patterns and the sense of entitlement that characterize the lives of the top 20% of the world must change radically. However, insufficient emphasis is given to how crucial it is to talk about these processes. Unless there is widespread reversal of such denial, developing sustainability will be a pipe dream in the face of the extensive but potentially reversible, frivolous and wasteful use of limited resources that threaten our collective future and the resilience of our planet.^25,26^

It is also crucial to more widely acknowledge exploitation of the weak and poor by the powerful and wealthy that has greatly benefitted about 20% of the world’s population over the past 40 years (in the process widening health disparities), and that resources continue to be stripped from the bottom end of the top 20% and shifted upwards.^27^ It was recognized many decades ago that with diminishing exploitation of other countries by the United States, there would be a shift of exploitative processes from external to internal sources.^28^ The impact on health and health care in the United States is illustrative.^27^ Galbraith also perceived such adverse effects of the trajectory of progress within an affluent society characterized by overblown expectations.^29^ Oxfam^30^ and the Pew Research Center have provided the details of increasingly adverse distributional changes globally.^31^

It is gratifying to see concerns expressed that ‘much of the discourse about solidarity and reducing inequality sounds shallow and hollow,’ and that it is ‘difficult to challenge currently dominant orthodoxies on global inequality and climate change.’ These concerns implicitly acknowledge the need to expose and vigorously contest the incoherence, hypocrisy, fraud and neglect of deep ethical considerations that are so central to many feel-good, window-dressing discourses associated with the neoliberal global political economy.^32,33^

Ooms and colleagues describe three challenging solutions conceived within the realm of current thought and then consider some imaginative solutions, despite their political infeasibility in an era when political change (Trump, Brexit etc) is taking the world in the opposite direction. Their optimistic conclusion, that there is possibly a small window of opportunity and that some ‘*real* out of the box thinking’ is necessary to take this opportunity, is deserving of support. Yet the ‘concerted and united advocacy of global health activists, human rights activists and trade unions’ that they call for will need even more imaginative support, given that global health activism is currently dominated by a biomedical model of health, human rights activism is largely (but not entirely) limited to civil and political rights, and trade unions seem self-serving and parochial. So their recommendations, like those of the Lancet-Oslo Commission, remain weak.^17^

David McCoy gets to the heart of the problem in concurring that a more critical approach to global health is required – one that goes beyond the popularly accepted belief that progress and future solutions can be achieved merely through more neoliberal development, technological advancement and philanthropy.^34^ I fully endorse his advocacy ‘for the global health community to: (*i*) create more space for social and political sciences, within global health, (*ii*) be prepared to act politically and challenge power, and (*iii*) do more to bridge the global-local divide in recognition of the fact that progressive change requires mobilization from the bottom-up in conjunction with top down policy and legislative change.’ McCoy’s call resonates with criticisms of philanthrocapitalism,^7^ previous calls for new Grand Challenges,^35^ and the need for new paradigms of thinking and action through co-operative transdisciplinary work.^36,37^ Our value system should be vigorously indicted for inadequately funding research into innovative and effective ways of promoting and applying such vital endeavors as those described by McCoy and others before him.

In the same way that the complexity of research towards developing an HIV vaccine involves considerable multi-disciplinary and transdisciplinary work, complex global health problems are also deserving of innovative means of finding and implementing new ameliorative societal pathways. The urgency of this task is enhanced by widening economic disparities in Europe, the United States and elsewhere, that have long been the predictable outcome of 40 years of neoliberal economic policies.^38,39^ By arguing for opposition to such policies, Ron Labonté’s reminder that *we already know what to do but cannot do this,* recalls the insight that *“The only thing necessary for the triumph of evil is that good men do nothing.*”^40^ He is thus supportive of strong and sustained left populism to be built in part on an ecocentric framework. It is acknowledged that achieving this poses almost insurmountable challenges, but also that this does not diminish the need to try.

Ron Labonté, David McCoy, and Gorick Ooms share with me some cautious optimism that, given human ingenuity and much public (but sadly not political) good will, these adverse social and economic forces could possibly be counteracted. Yet it is perhaps more realistic to understand that human nature is such that this is unlikely, as illustrated by our tardiness in responding to what has been obvious for so long, and with the proverbial half-full glass heading for three quarters empty!^41^ For example, in 1932, Aldous Huxley’s *Brave New World* predicted a dystopic world several centuries into the future.^42^ By 1958 such change was surprisingly already becoming evident.^43^ For example, Huxley noted that by 1958 freedom was on the decline even in those countries with a tradition of democratic government, and the shift toward totalitarian forms of control was advanced far beyond that projected in the 1930s. Commercial and political organizations had already developed new techniques for manipulating the thoughts and feelings of the masses, and population growth was beginning to outstrip food production in poor countries. Such socio-economic trends have intensified in recent decades and attention is being refocused on dystopic scenarios.^44^

Given the unsatisfactory and unpredictable nature of progress,^45^ and the critical state of the world,^46^ ongoing consideration of alternative possibilities for better social systems continues.‘Imperial common sense’ should be challenged^47^ and widespread support generated for use of our capacity to do better for global/planetary health through ‘rethinking the traditional bureaucratic model of postwar intergovernmental organizations.’^48^ A much more ambitious agenda is needed for transdisciplinary research and collaborative activism, together with revival of the ‘globalization from below movement,’ and promotion of innovative conversations embracing new paradigms, that could possibly act as much-needed catalysts for change.

## Ethical issues

Not applicable.

## Competing interests

Author declares that he has no competing interests.

## Author’s contribution

SB is the single author of the paper.
